# Efficiency of pulsed electromagnetic fields on pain, disability, anxiety, depression, and quality of life in patients with cervical disc herniation: a randomized controlled study

**DOI:** 10.3906/sag-1901-65

**Published:** 2019-08-08

**Authors:** Erkam HATTAPOĞLU, İbrahim BATMAZ, Banu DİLEK*, Mehmet KARAKOÇ, Serda EM, Remzi ÇEVİK

**Affiliations:** 1 Department of Physical Medicine and Rehabilitation, Faculty of Medicine, Dicle University, Diyarbakır Turkey; 2 Department of Physical Medicine and Rehabilitation, Faculty of Medicine, Dokuz Eylül University, İzmir Turkey

**Keywords:** Pulsed electromagnetic fields, cervical disc herniation, pain, quality of life

## Abstract

**Background/aim:**

In this study, it was aimed to investigate the effects of****pulsed electromagnetic field****(PEMF) therapy on pain, disability, psychological state, and quality of life in cervical disc herniation.

**Materials and methods:**

Patients were randomly divided into two groups, including Group 1, which received a therapy consisting of transcutaneous electrical nerve stimulation (TENS), hot pack (HP), and PEMF, and Group 2, which received a magnetic field (sham magnetic field) without current flow in addition to TENS and HP therapy. Pain was assessed by a visual analog scale (VAS, 0–10 cm). The other outcome measures were function (Neck Pain and Disability Scale), anxiety-depressive mood (Hospital Anxiety and Depression Scale), and quality of life (Nottingham Health Profile). All evaluations were performed at baseline, in the 3rd week, and in the 12th week after treatment.

**Results:**

A significant improvement was found in the neck pain, disability, depression, anxiety, and quality of life scores of both groups after treatment when compared to those before treatment. However, in the comparison between changes within groups, significant improvements were determined only in the VAS and Nottingham Health Profile sleep subparameter in the 12th week after treatment compared to those before treatment.

**Conclusion:**

PEMF therapy in cervical disc herniation can be used safely in routine treatment in addition to conventional physical therapy modalities.

## 1. Introduction

Neck pain leads to functional limitations and inadequacies by affecting the daily life activities of individuals negatively [1,2]. Cervical disc herniation (CDH) is one of the significant causes of neck pain, which occurs as a result of the nucleus pulposus leaking out of the annulus fibrosis, which is ruptured for various reasons, and creating pressure on the spinal cord and nerve roots [3]. 

Pulsed electromagnetic field (PEMF) therapy is a noninvasive, inexpensive, and safe physical therapy agent with no known significant side effects. Its beneficial effects in many musculoskeletal diseases, such as fracture healing, nerve regeneration, osteoarthritis, fibromyalgia, and osteoporosis, have been revealed [4–8].

PEMF therapy has been demonstrated to be effective in the treatment of many diseases, especially locomotor system diseases, because of its antiinflammatory, antiedema, analgesic, antispasmodic, and blood-boosting effects [9–12]. PEMF therapy has been reported to show these effects through its regulatory effects on the stimulation of lysosomes, hormone secretion, regulation of enzymatic activities, increase of DNA and collagen synthesis, regulation of calcium metabolism, receptor modification and membrane permeability, and materials such as adenylyl cyclase, cAMP, and protein kinase [9–11]. 

There are limited studies evaluating the effects of PEMF therapy on pain and disability in cases of mechanical neck pain and cervical osteoarthritis. However, we did not encounter any study evaluating the efficacy of PEMF therapy only in the treatment of CDH. In the present study, it was aimed to investigate the efficacy of PEMF therapy on pain, disability, psychological state, and quality of life in CDH.

## 2. Materials and methods

In this prospective, placebo-controlled, randomized double-blind study, volunteer patients between 18 and 65 years of age diagnosed with CDH, who were admitted to the Dicle University Faculty of Medicine Physical Medicine and Rehabilitation Outpatient Clinic between September 2016 and August 2017 with the complaint of neck and arm pain for more than 2 months and in whom intervertebral disc protrusion or extrusion was detected in magnetic resonance imaging (MRI) of the cervical region, were included. Before the study, approval was obtained from the Ethics Committee of the Dicle University Faculty of Medicine. All the recruited subjects signed an informed consent form before participating in the study. 

Patients who had received physical therapy within 3 months, those using analgesics or nonsteroidal antiinflammatory drugs, those with neurological deficits, and patients who were diagnosed with cervical myelopathy or cervicobrachial syndrome were excluded from the study. Furthermore, patients with previous cervical surgery, with inflammatory diseases such as rheumatoid arthritis or ankylosing spondylitis, and those with osteoporosis, fibromyalgia, myofascial pain syndrome, thoracic outlet syndrome, cancer, chronic liver disease, chronic kidney disease, chronic heart disease, or psychiatric diseases were also not included in the study. Female patients who might have been pregnant were also excluded from the study. 

The detailed anamnesis of the patients was obtained, and their demographic characteristics were recorded. Complete blood count, erythrocyte sedimentation rate, serum C-reactive protein (CRP), and routine biochemical analyses were determined.

The present study was designed as a prospective, double-blinded randomized controlled trial with three measurement points (baseline, 3rd week, and 12th week after treatment). Seventy-four patients who met the eligibility criteria were randomly allocated to either the intervention group (Group 1) or control group (Group 2). Randomization was applied with a simple random approach by using a table of random numbers.

 Group 1 received therapy consisting of transcutaneous electrical nerve stimulation (TENS), a hot pack (HP), and PEMF. Group 2 received a magnetic field (sham magnetic field) without current flow in addition to TENS and HP therapy.

The patients did not know what treatment they were receiving. The treatments were applied by the same technician. The evaluation of the patients was performed by the same physician, who did not know which groups patients were in.

HP and TENS were applied together for 20 min. TENS was applied to the paravertebral muscles by a dual-channel Chattanooga Intelect Advanced Monochromatic Combo electrotherapy device with two carbon electrodes, and the highest level of current that the patient could tolerate was delivered in the conventional mode (frequency 100 Hz, current duration 40 ms). 

PEMF therapy was applied to the cervical region by ASA EASY Quattro PRO (Arcugnano, Italy). Magnetotherapy was applied at low frequency (50 Hz), with intensity of 0.6 mT and application time of 20 min. The control group (Group 2) received a magnetic field (sham magnetic field) without current flow in addition to TENS and HP therapy. This therapy program was implemented 5 days a week for 3 weeks.

In both groups, the use of analgesics was not allowed, except for a maximum of 2000 mg/day of paracetamol.

A visual analogue scale (VAS) was used to determine the severity of pain. The VAS is a 10-cm line; the left end indicates no pain while the right end indicates intolerable pain [13].

Pain and related disability during the daily life activities of the patients were assessed by the Neck Pain and Disability Scale (NPDS), the reliability and validity of which were tested in Turkish people [14]. The NPDS consists of a 20-item questionnaire addressing neck problems, pain intensity, emotion and cognition, and interference with life activities. Each item is scored between 0 and 5 (0: no pain or activity limitation, 5: as much pain as possible or maximal limitation), and the maximum score is 100. Higher scores indicate a worse quality of life [15].

The Hospital Anxiety and Depression (HAD) scale was used to measure the depression and anxiety levels of the patients. The HAD scale is a 14-item questionnaire. The questionnaire comprises seven questions for anxiety and seven questions for depression (score range: 0–21). Seven was found to be the cut-off score for the depression subscale and 10 for the anxiety subscale [16,17]. The validity and reliability studies of the Turkish version of the HAD scale were performed by Aydemir et al. [17].

The patient’s quality of life was assessed by the Nottingham Health Profile (NHP) questionnaire. This scale is a 38-item questionnaire with 6 subareas, and it evaluates energy level, pain, emotional reaction, sleep, social isolation, and physical abilities. Each question is answered by “yes” or “no” with each question assigned a weighted value. The items are scored from 0 to 100. Higher scores indicate a worse quality of life. The validity and reliability studies of the Turkish version of the NSP were performed by Küçükdeveci et al. [18].

### 2.1. Statistical analysis

The data were analyzed using SPSS 18 (SPSS Inc., Chicago, IL, USA). The Kolmogorov–Smirnov test was used to test for normality. Quantitative variables are presented as mean (x) ± standard deviation (SD), whereas categorical variables are presented as number and %. Comparisons between the groups were made by the independent-samples t-test or Mann–Whitney U test according to the compatibility of the data with normal distribution. The difference between proportional variables was calculated by the chi-square test. Changes over time of more than two measurements in the groups were calculated by the repeated measures analysis of variance method. The hypotheses were bidirectional, and P ≤ 0.05 was considered statistically significant.

## 3. Results

The patients were recruited from September 2016 to August 2017. Of the 140 patients, 42 patients did not meet the inclusion criteria, and 3 patients did not agree to participate in the study. The study included 74 patients. Five patients in each group dropped out for different reasons. Thus, 64 patients completed the study. The Consolidated Standards of Reporting Trials (CONSORT) flow diagram of the study is presented in the Figure.

**Figure:**
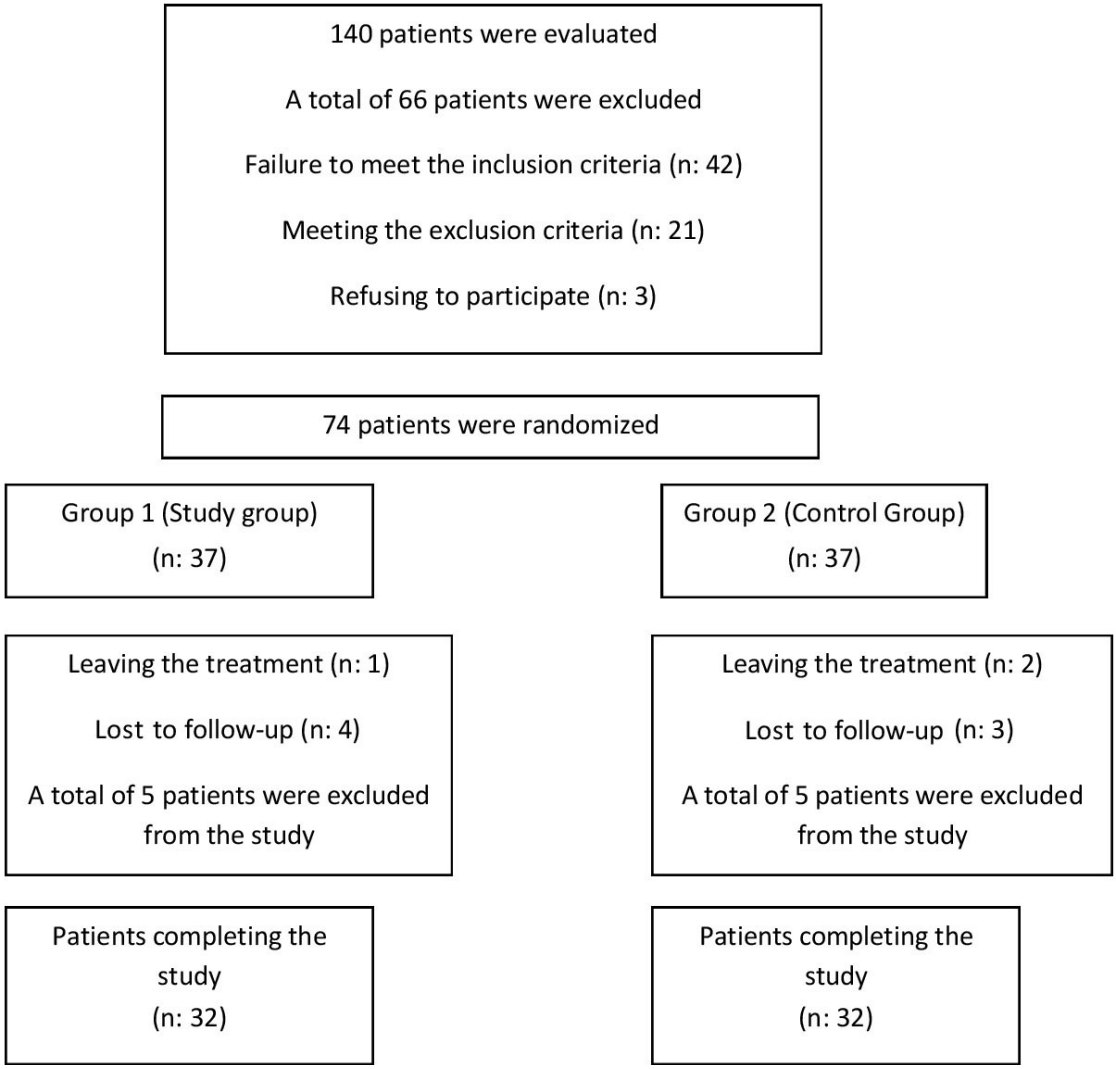
Patient flow chart

### 3.1. Demographic data

There were 22 (68.8%) females and 10 (31.3%) males in Group 1, and 24 (75%) females and 8 (25%) males in Group 2. The demographic characteristics of Group 1 and Group 2 are listed in Table 1.

**Table 1:** The demographic characteristics of the groups (mean ± SD or n, %).

	Group 1 (n = 32)	Group 2 (n = 32)	P
Age (years)	42.96 ± 10.35	47.15 ± 11.03	0.12
Sex (male, %)	10 (31.3)	8 (25)	0.57
BMI (kg/m2)	28.29 ± 4.95	27.38 ± 4.47	0.44
Symptom duration (years)	3.58 ± 5.52	3.50 ± 3.83	0.54

When the groups were compared in terms of the size of the disc herniation, there was no significant difference between the groups. While 30 patients had protruded herniation and two patients had extruded herniation in Group 1, 27 patients had protruded herniation and five patients had extruded herniation in Group 2 (P = 0.23).

### 3.2. Pain and function

There was no significant difference in terms of the VAS (0–10 cm) pain and NPDS function scores before treatment in both groups. Significant improvement was observed in both groups in week 3 and week 12 after treatment. Upon comparing the groups, there was no significant difference in terms of the NPDS scores, and when the changes within the groups were compared in terms of VAS in week 12, it was indicated that there was a significant improvement in Group 1 (Table 2).

**Table 2:** Pain and function of groups.

	Group 1 (n = 32)	Group 2 (n = 32)	P (MWU)
Pain (VAS 0–10 cm)Baseline3rd week12th week	pA 7.09 ± 1.67 3.62 ± 2.59 † <0.001*4.78 ± 2.80 ‡	pA7.37 ± 1.694.43 ± 2.39 † <0.001* 6.25 ± 2.44 ‡	0.570.190.02*
Change (0–12)	2.31 ± 2.30	1.12 ± 1.99	0.03*
Function (NPDS)	pA	pA	
Baseline	60.25 ± 17.01	61.12 ± 19.47	0.84
3rd week	42.03 ± 21.58 † <0.001*	44.53 ± 20.81 † <0.001*	0.63
12th week	48.37 ± 22.30 ‡	54.34 ± 20.74 ‡	0.27

MWU: Mann–Whitney U test, A: ANOVA, †: baseline - 3rd week, ‡: baseline - 12th week differences within groups (Wilcoxon signed ranks test/paired samples test), * P < 0.05.

### 3.3. Anxiety and depression

There was no significant difference between the groups in the HAD-Anxiety and Depression Scale scores before treatment. In the third week after treatment, the HAD anxiety scores significantly improved in both groups; however, a significant difference was found only in Group 1 in the 12th week after treatment. There was no significant difference between the two groups in the 3rd and 12th weeks when the groups were compared (Table 3). While a significant improvement was found in the HAD depression scores in Group 1 in the 3rd and 12th weeks after treatment, no significant difference was found in Group 2 (Table 3).

**Table 3:** Anxiety-depression and moods of groups.

	Group 1 (n = 32)	Group 2 (n = 32)	P (MWU)
Anxiety-depression MoodHAD-AnxietyBaseline3rd week12th week	pA7.59 ± 3.426.46 ± 2.91† 0.006*6.78 ± 2.77 ‡	pA 7.50 ± 3.77 6.53 ± 3.13 † 0.01* 6.87 ± 3.46	0.910.930.90
HAD-Depression	pA	pA	
Baseline	7.56 ± 2.63	7.75 ± 3.21	0.79
3rd week	6.65 ± 3.38 † 0.03*	7.06 ± 3.55 0.08	0.64
12th week	6.87 ± 3.03 ‡	7.43 ± 3.11	0.69

MWU: Mann–Whitney U test, A: ANOVA, †: baseline - 3rd week, ‡: baseline - 12th week differences within groups (Wilcoxon signed ranks test/paired samples test), * P < 0.05.

### 3.4. Quality of life

Before treatment, there was no significant difference between the two groups in terms of all NHP subparameters, except for the NHP total score. While there was a significant improvement in the pain, physical activity, energy, sleep, and total NHP scores in Group 1 in the 3rd and 12th weeks after treatment, there was a significant improvement in pain and emotional reaction scores in Group 2 (Table 4). 

**Table 4:** Quality of life in groups.

NHP	Group 1 (n = 32)	Group 2 (n = 32)	P (MWU)
NHP-PBaseline3rd week12th week	pA63.59 ± 28.7838.93 ± 34.76 † <0.001*47.28 ± 35.01‡	pA71.12 ± 32.2953.21 ± 36.63† <0.001*62.59 ± 32.88‡	0.320.110.07
NHP-ER			
Baseline	30.59 ± 27.89	41.71 ± 35.50	0.16
3rd week	21.50 ± 23.85† 0.01	34.37 ± 35.48 0.007*	0.09
12th week	25.56 ± 25.42	38.50 ± 36.03	0.10
NHP-S			
Baseline	33.71 ± 32.62	46.90 ± 34.94	0.12
3rd week	17.78 ± 26.90† 0.001*	39.12 ± 32.59 0.05	0.006*
12th week	22.71 ± 28.27‡	45.53 ± 32.69	0.004*
Change (0–12)	11.00 ± 23.09	1.37 ± 12.66	0.04*
NHP-SI			
Baseline	18.93 ± 24.18	33.96 ± 38.95	0.06
3rd week	10.31 ± 18.09 0.08	25.84 ± 35.78† 0.007*	0.03*
12th week	17.18 ± 25.83	35.15 ± 39.18	0.03*
NHP-PA			
Baseline	27.78 ± 19.87	40.84 ± 30.33	0.05
3rd week	17.37 ±17.56† 0.001*	36.96 ± 32.15 0.25	0.04*
12th week	22.06 ± 19.25‡	40.09 ± 28.49	0.004*
NHP-EL			
Baseline	58.12 ± 34.58	72.34 ± 32.51	0.09
3rd week	39.03 ± 32.87† 0.001*	66.43 ±36.95 0.37	0.003*
12th week	49.90 ± 36.74‡	69.90 ±32.33	0.02*
NHP-T			
Baseline	233.5 ± 120.6	306.9 ± 164.1	0.04*
3rd week	144.1 ± 116.1† <0.001*	253.1 ± 172.3† 0.001*	0.004*
12th week	183.8 ± 128.3‡	292.1 ± 162.6	0.004*

MWU: Mann–Whitney U test, A: ANOVA, †: baseline - 3rd week, ‡: baseline - 12th week differences within groups (Wilcoxon signed ranks test/paired samples test), * P < 0.05. P: Pain, ER: emotional reaction, S: sleep, SI: social isolation, PA: physical activity, EL: energy level, T: total.

While there was no significant difference in terms of social isolation in Group 1 in the 3rd and 12th weeks after treatment, there was no significant difference in sleep, physical activity, and energy subparameters in Group 2 (Table 4). 

When the 2 groups were compared, while physical activity, energy level, and total scores in the NHP significantly improved in Group 1, social isolation and emotional reaction were significantly improved in Group 2 at 3 and 12 weeks. When the changes within the groups were compared, a significant improvement was observed in the sleep subparameter in Group 1 only in the 12th week after treatment when compared to that before treatment. No significant difference was found in the comparison of other parameters (Table 4).

## 4. Discussion

In this study, while the addition of PEMF therapy to the conventional physical therapy program in patients with CDH provided an improvement in pain and sleep in the 12th week, its additional contribution to function, general quality of life, anxiety, and depression could not be demonstrated.

In a study evaluating the efficacy of PEMF therapy on pain, joint range of motion, and functional status in cervical osteoarthritis patients, significant improvements were found in pain, muscle spasm, joint movements, and NPDS scores in the treatment group compared to the control group [19]. In another placebo-controlled study in which the efficacy of PEMF therapy was evaluated in patients with chronic neck pain, a significant improvement in neck pain and joint range of motion was observed in patients receiving PEMF therapy compared to the control group. Furthermore, it was stated that PEMF therapy could be used easily in the treatment of chronic neck pain due to the absence of side effects [20]. Similarly, in our study, significant improvements were found in the pain scores of the patients between the period before treatment and the 12th week after treatment compared to the control group. This result may indicate that the effects of PEMF therapy appear in the late period. It is possible to attribute this late effect to the mechanisms of action of PEMF therapy (stimulation of lysosomes, hormone secretion, regulation of enzymatic activities, increase of DNA and collagen synthesis, regulation of calcium metabolism, receptor modification and membrane permeability, regulation of materials such as adenylyl cyclase, cAMP, protein kinase) [9–11].

In a study carried out by Lee et al., the effects of the electromagnetic field on the proliferation of human intervertebral disc (IVD) cells were investigated, and the electromagnetic field was found to stimulate DNA synthesis in IVD cells. Thus, the researchers concluded that the electromagnetic field could be used to stimulate the proliferation of IVD cells in the cellular treatment of degenerative disc disease [21]. In another study, it was emphasized that PEMF therapy had significant impacts on the expression of genes associated with the early stages of inflammation and some effects on genes associated with matrix degradation. The effects of PEMF on proinflammatory cytokine and MMP expression highlight a potential role of PEMF in the treatment of inflammation in IVDs. Moreover, the authors concluded that IVD cells are responsive to PEMF and future studies are warranted to determine whether PEMF may be helpful for patients with IVD degeneration [22]. In our study, we can explain the positive effects of PEMF therapy on pain in patients with CDH by these mechanisms.

Neck pain leads to functional limitations and disabilities by influencing physical and psychological functions. Moreover, it causes difficulties in daily life activities [1,23]. In the evaluation made within Group 1, a significant decrease was found in both the anxiety and depression scores before and after treatment and in the 12th week after treatment. However, there was no significant difference in the HAD scores between the two groups in the comparison between the groups in differences before and after treatment. In the study carried out by Boskovic et al., it was found that PEMF and laser treatments did not cause heat and electric sensation in patients, and therefore they were not very good at eliminating psychoneurotic symptoms compared to other treatment methods, but they were effective on vascular or neurological cervical syndromes [24]. Similarly, one of the reasons for the absence of a significant difference between the groups in our study may be that PEMF therapy does not create any felt sense in patients. 

Chronic neck pain not only causes weakness of the neck muscles but also decreases the quality of life [25]. In our study, in the evaluation made within Group 1, a significant improvement was observed in all parameters, except for the NHP-social isolation and NHP-emotional reaction subparameters. Furthermore, in the evaluation between the changes within groups, an improvement only in the NHP-sleep subparameter was found to be significant in the 12th week after treatment compared to that before treatment. Poor sleep quality is strongly related to chronic pain [26]. As a result, a decrease in pain in the 12th week after treatment in our patients may have contributed positively to sleep.

The present study had certain limitations. The first limitation of the study is the assessment of short-term but not long-term effects of treatment. The second one is the usage of TENS and HP therapies together and not evaluating the effect of PEMF therapy alone since they have similar efficacy. The third one is the limited patient number and sample size not being calculated in this study. Although during the study the patients were allowed to use paracetamol up to a maximum of 2000 mg/day when needed, the total paracetamol dose used by the patients was not recorded. In conclusion**, **PEMF therapy in CDH can be used safely in routine treatment in addition to conventional physical therapy modalities due to the relatively late onset of the effect. A significant improvement was found in the neck pain, disability, depression, anxiety, and quality of life scores of both groups after treatment when compared to those before treatment. Furthermore, in the comparison between the changes within groups, a significant improvement was observed in the pain and NHP-sleep subparameter in the 12th week after treatment when compared to those before treatment. Since parameters such as intensity, frequency, and frequency of application applied in PEMF therapy differ in many studies, randomized controlled studies are needed to standardize these parameters.
